# Bacterioplankton community shifts associated with epipelagic and mesopelagic waters in the Southern Ocean

**DOI:** 10.1038/srep12897

**Published:** 2015-08-10

**Authors:** Zheng Yu, Jun Yang, Lemian Liu, Wenjing Zhang, Stefano Amalfitano

**Affiliations:** 1 Aquatic EcoHealth Group, Key Laboratory of Urban Environment and Health, Institute of Urban Environment, Chinese Academy of Sciences, Xiamen 361021, P. R. China; 2 Marine Biodiversity and Global Change Research Center, College of Ocean and Earth Sciences, Xiamen University, Xiamen 361102, P. R. China; 3 Water Research Institute, National Research Council of Italy (IRSA-CNR), Monterotondo, Roma, 00015, Italy

## Abstract

The Southern Ocean is among the least explored marine environments on Earth, and still little is known about regional and vertical variability in the diversity of Antarctic marine prokaryotes. In this study, the bacterioplankton community in both epipelagic and mesopelagic waters was assessed at two adjacent stations by high-throughput sequencing and quantitative PCR. Water temperature was significantly higher in the superficial photic zone, while higher salinity and dissolved oxygen were recorded in the deeper water layers. The highest abundance of the bacterioplankton was found at a depth of 75 m, corresponding to the deep chlorophyll maximum layer. Both Alphaproteobacteria and Gammaproteobacteria were the most abundant taxa throughout the water column, while more sequences affiliated to Cyanobacteria and unclassified bacteria were identified from surface and the deepest waters, respectively. Temperature was the most significant environmental variable affecting the bacterial community structure. The bacterial community composition displayed significant differences at the epipelagic layers between two stations, whereas those in the mesopelagic waters were more similar to each other. Our results indicated that the epipelagic bacterioplankton might be dominated by short-term environmental variable conditions, whereas the mesopelagic communities appeared to be structured by longer water-mass residence time and relative stable environmental factors.

The Southern Ocean has unique oceanographic dynamics with major implications for the global ocean circulation and water stratification system^1^. The frequent sea ice freeze-thaw cycles create cold and salty waters which sink to the seafloor, generating vertical environmental gradients along the water column in terms of temperature, salinity, and dissolved oxygen^2^. How such variations influence the biological community is still an open question. In particular, little is known about the patterns and drivers of microbial diversity in the Southern Ocean, although microbes are ubiquitous in the ocean and may comprise up to 70–75% of the total plankton biomass^1^. This lack of knowledge holds true also for planktonic bacteria, owing to a combination of reasons such as limited sampling efforts and methodological constraints^3^.

Despite recent improvements in molecular biological methods (e.g., high-throughput sequencing) which can help reveal the massive taxonomic microbial richness^4^, the spatial patterns and structure of bacterial communities (e.g., occurrence and proportions of different phylogenetic groups) are not well explored in the Southern Ocean compared with marine waters from tropical, temperate and other polar regions^1^,^5^,^6^. Previous studies have reported the biogeographical distribution of some microbial groups in Southern Ocean surface waters at different levels of phylogenetic resolution^7^,^8^. Others showed that spatiotemporal differences can be detected in the structure of bacterioplankton communities^9^,^10^. In general, some recurrent patterns in microbial community assemblages have been identified at a broad phylogenetic level. As in most marine regions, the dominant phylogenetic groups include Alphaproteobacteria, especially the SAR11 clade, Cyanobacteria, Gammaproteobacteria, and Bacteroidetes (e.g. Flavobacteria)^5^. In several instances, the distribution of marine bacteria has been linked with environmental parameters. For example, Abell and Bowman showed that the abundance of particle-associated Flavobacterium was positively correlated with seawater chlorophyll-a and nutrient concentrations^11^.

There appears to be a subtle interaction between inflows, water column stability, periodic overturns and bacterioplankton community composition in the Southern Ocean, as well as in other aquatic systems^12^,^13^,^14^. Only a few studies have explored the vertical microbial distribution in pelagic polar waters, and the spatial patterns of bacterial community structure and diversity can be explained in part by the local niche-based processes^15^,^16^,^17^. Ghiglione *et al.* reported that only 25% bacterioplankton taxa were common at the surface waters between the Southern and Arctic Oceans, and the coastal surface communities from both polar oceans exhibited more dissimilar from their respective open ocean communities due to local variability in environmental conditions; whereas the differences were not as pronounced among those microbial taxa at deep waters largely due to long water-mass residence time and connectivity through ocean circulation^15^. Since epipelagic waters are more susceptible to abiotic and biotic perturbations than deeper mesopelagic waters, we hypothesized that vertical patterns and diversity profiles of bacterial communities could reflect the different levels of environmental stability that clearly differentiate surface from deeper waters.

The aim of this study was to assess the overall variability in bacterial community structure along well-defined vertical gradients and between two adjacent locations of the Southern Ocean. This study will allow us a new perspective of how bacterioplankton communities change along the water column in terms of abundance and diversity, and how environmental gradients and historical processes control the overall variability of microbial communities in epipelagic and mesopelagic waters in the Southern Ocean.

## Materials and Methods

### Study site and sample collection

Water samples were collected during the Antarctic summer from two sampling sites located around 150 km apart from each other (station A - 47°10′ W, 61°30′ S - January 2012; station B - 44°41′ W, 61°29′ S - December 2011). Depth, water temperature, salinity, dissolved oxygen, and electrical conductivity were measured *in situ* with a XR-420 CTD profiler (RBR Ltd., Ottawa, ON, Canada). CTD salinity was calibrated following an inter-comparison with high-precision salinity measurements made with shipboard CTD instruments. Different sampling depths were selected in order to explore the entire water column in the transition from epipelagic to mesopelagic areas at both stations. Water samples for bacterial community analysis were collected using a closing Kemmerer bottle at nine depths ranging from 0 to 700 m in station A and from 0 to 350 m in station B, respectively. About 3 liters per sample were immediately filtered in triplicate through 0.22 μm pore size polycarbonate membranes (47 mm diameter, Millipore, Billerica, MA, USA) for DNA extraction. The membranes were placed into sterile 5 mL centrifuge tubes, and stored in a −80 °C freezer until molecular analyses. Total DNA was extracted directly from the membranes using a FastDNA spin kit (BIO101 systems, MP Biomedicals, Solon, OH, USA) according to the manufacturer’s instructions. Purified DNA was dissolved in 50 μl ddH_2_O and stored at −20 °C until use.

### Quantitative real-time PCR and standard curve

To assess bacterial 16S rRNA gene copy number as a proxy for bacterial abundance, we ran quantitative PCR on each sample of DNA in conjunction with primers 341F (5′- CCTACGGGNGGCWGCAG-3′) and 515R (5′-ATTCCGCGGCTGGCA-3′)^18^. Clone sequencing was first used to construct standard curves as follows: 50 μl PCR mixture containing 1 μl of the primer set (25 pmol each), 0.5 μl (1.25 U) of *Ex Taq* DNA polymerase (Takara Bio, Otsu, Shiga, Japan), 5 μl of *Ex Taq* buffer, 4 μl of deoxyribonucleotide triphosphate (dNTP) mixture (2.5 mM each), 100 ng of DNA template, and 33.5 μl of ddH_2_O. PCR was performed in the following thermal cycles: initial denaturation at 94 °C for 5 min; 25 cycles at 94 °C for 30 s, 51 °C for 30 s and 72 °C for 60 s; and a final extension at 72 °C for 10 min. The purified PCR products were ligated into the pMD18-vector (Promega, Madison, WI, USA) and transformed into *Escherichia coli* DH5α competent cells (Takara Bio, Otsu, Shiga, Japan). The plasmids containing 16S rRNA gene fragments were sequenced using an automated ABI3730 DNA Sequencer (Applied Biosystems, Foster City, CA, USA). Successfully inserted plasmid DNA was then extracted using the MiniPrep kit (QIAGEN GmbH, Hilden, Germany) and plasmid concentrations were determined by spectrophotometry using a BioPhotometer (Eppendorf, Hamburg, Germany). A standard curve was prepared from linearized plasmid serial dilutions containing 10^4^ and 10^10^ 16S rRNA genes copies calculated directly from the concentration of extracted plasmids. The curve was generated by plotting the threshold cycle values versus log10 of the gene copy numbers. The amplification efficiency (*E*) was estimated using the slope of the standard curve through the following formula: *E* = (10^−1/slope^) − 1. The reasonable efficiency of the PCR should be between 95% and 105%^19^.

Quantitative PCR amplification was performed from known concentrations of template DNA to construct standard curves for the quantification of environmental samples. PCR amplification of 16S rRNA genes was performed in triplicate on an Applied Biosystems 7500 Real-Time PCR System (Applied Biosystems, Foster City, CA, USA). The quantitative PCR assay was carried out in a volume of 20 μl including 10 μl SYBR Premix *Ex Taq*^™^, 0.5 μM of each primer (341F and 515R), 2 μl of total DNA, and RNase-free water. The thermocycling steps of the quantitative PCR were according to the manufacturer’s instructions (Takara Bio, Otsu, Shiga, Japan). All the measurements were performed in triplicate and no signal was detected in our no template control (NTC) samples. In this study, the *R*^2^ value for linear regression of threshold value and standard abundance was 0.992, indicating that the assays were quantitative across the range of 16S rDNA concentration tested. The amplified products were visualized on a Gel Documentation system (BioRad, Hercules, CA, USA) after running in 1% agarose gel.

### High-throughput sequencing and sequence analysis

The hypervariable V4 region (~207 bp) of the 16S rRNA gene was amplified using the primers: 16S-F (5′-AYTGGGYDTAAAGNG-3′) and 16S-R (5′-TACNVGGGTATCTAATCC-3′)^20^. Sequencing was performed on an Illumina MiSeq instrument using a paired-end 150-bp sequence read run with the Miseq Reagent Kit v3 at the Personal Biotechnology Company (Shanghai, China). Each DNA sample was individually PCR-amplified in triplicated 25-μL reactions. The cycling conditions were an initial denaturation at 94 °C for 5 min, followed by 25 cycles of 94 °C for 30 s, 50 °C for 30, 72 °C for 30 s, and a final 7 min extension at 72 °C. Each reaction contained 1 × PCR buffer, 2.5 mM dNTPs, 0.625 U of *Taq* DNA polymerase (Takara Bio, Otsu, Shiga, Japan), 10 μM of each primer, and 20 ng of target DNA. Sequences were processed by using MOTHUR v.1.20.1^21^. Briefly, any sequences of length <150 or >300, mean quality <30, ambiguous bases >1, homopolymer length >6, maximum primer mismatch >0 were removed from further analysis^22^. The remaining sequences were aligned to a reference alignment, and those sequences that did not align to the correct region were eliminated. Further, any non-bacterial ribosome sequences and chimeras were removed using Black Box Chimera Check software (B2C2) according to a previously used procedure^23^. Sequences were split into groups according to their taxonomy and assigned to operational taxonomic units (OTUs) at a 3% dissimilarity level. OTUs containing 1 read (singleton) were not used to avoid or at least reduce possible sequencing errors. For the following data analyses, we used a randomly selected subset of 27582 sequences from each sample to standardize sequencing effort across samples. We then used Bayesian classifier to classify those sequences against the Ribosomal Database Project (RDP) 16S rRNA gene training set (version 9, http://rdp.cme.msu.edu)^24^. We required an 80% pseudobootstrap confidence score^25^. All Archaea, Eukaryota, chloroplasts and mitochondria were culled^22^. BLAST searches based on the NCBI GenBank database (www.ncbi.nlm.nih.gov/blast) were also performed to provide information on the OTUs that were not classified by RDP. Dominant OTUs were defined as those with a representation ≥5% within a sample, abundant OTUs were defined as those with a representation ≥1% within a sample, and rare OTUs were defined as having an abundance <0.01% within a sample, which corresponds to the definitions of Ghiglione *et al.*^15^, Liu *et al.*^22^ and Galand *et al.*^26^. No successful PCR-amplification for high-throughput sequencing was observed in stations A 500 m, 700 m and stations B 150 m, 200 m and 300 m samples in this study, thus only 13 samples were included in downstream analyses.

### Data analysis

The Shannon-Wiener index (*H’*) was calculated for the 16S rRNA gene diversity. The *H’* was determined with the following equation: *H’* = −Σ*P*_*i*_ln*P*_*i*_. The term *P*_*i*_ was calculated as follows: *P*_*i*_ = *n*_*i*_/*N*, where *n*_*i*_ is the number of sequences of the *i*^th^ OTU and *N* is the number of sequences of all OTUs in a community^27^. Cluster analysis was used to investigate differences in bacterial communities based on the entire, abundant and rare OTUs with the program PRIMER 5.0^22^. The bacterial abundance was log(x + 1) transformed to improve normality and homoscedasticity, and then a group-average linked method based on Bray-Curtis similarity coefficient was used in the cluster analysis^28^.

In order to discriminate communities in surface waters from those retrieved in deeper (more salty and oxygenated) waters, the samples were clustered into three groups (Group 1 - surface waters of station A from 0 to 100 m; Group 2 - surface waters of station B from 0 to 75 m; Group 3 - deeper waters from stations A and B). Thus, the similarity percentage (SIMPER) procedure was used to identify those species (or OTUs) that contributed most to the similarity and dissimilarity among the three groups^29^. Analysis of variance (ANOVA) was used in combination with Scheffe’s F multiple-comparison test to examine differences in the environmental and bacterial parameters among these three groups. Sequences of dominant (greater than or equal to 5%) OTUs were compared with sequences available in the GenBank database using BLASTN.

The normality of the environmental variables and bacterial abundance was checked using the Shapiro-Wilk test and variables were log(x + 1) transformed to improve normality and homoscedasticity for multivariate statistical analyses. The gradient length of the longest axis explored by detrended correspondence analysis (DCA) for microbial communities was longer than 3 SD (standard deviation) units, thus further analyses were conducted using a unimodal response model. Canonical correspondence analysis (CCA) was employed to evaluate the effect of environmental factors (including water temperature, salinity, dissolved oxygen and water depth) on the bacterial community structure. To evaluate the significance of the conditional effects, Monte Carlo permutation of full models was applied with 999 unrestricted permutations. There exist the possibility of collinearity among salinity and electrical conductivity, therefore only salinity was reserved for the CCA analysis. The above analyses were performed using the R software package with the vegan library (R Development Core Team).

## Results

### Vertical variations of abiotic factors and bacterioplankton abundance and diversity

**Figure 1:**
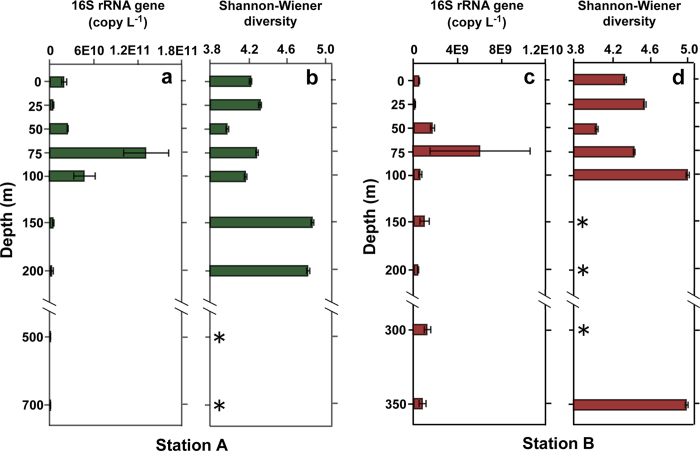
Vertical profiles of 16S rRNA gene copy number (a,c) and Shannon-Wiener diversity (b,d) of bacterial community from two Southern Ocean stations. *indicate missing data from the Illumina sequencing platform.

Water temperature was significantly higher in the epipelagic (Groups 1 and 2) than deeper waters (Group 3) in which higher values of salinity and dissolved oxygen were recorded (Table 1). The copy numbers of 16S rRNA genes at both stations A and B showed a similar stratified trend along the water column, and maximal values at both stations were detected at 75 m with an average copy number of 1.31 × 10^11^ L^−1^ and 6.18 × 10^9^ L^−1^, respectively (Fig. 1). The bacterial 16S rRNA gene copy number in station A rapidly decreased from 75 m to the deep waters and reached a minimal value (1.62 × 10^8^ copies L^−1^) at the 700 m layer. However, the lowest gene copy number value in station B was found at 25 m, with the copy number of 1.49 × 10^8^ L^−1^. Overall, the average abundance of 16S rRNA genes in the surface epipelagic waters was higher than that in deeper mesopelagic waters in station A. However, both number of OTUs and Shannon-Wiener diversity showed an opposite trend at both stations because they increased with water depth and showed a significant difference between surface and deep waters (Table 1).

### Patterns of bacterial community composition

**Figure 2:**
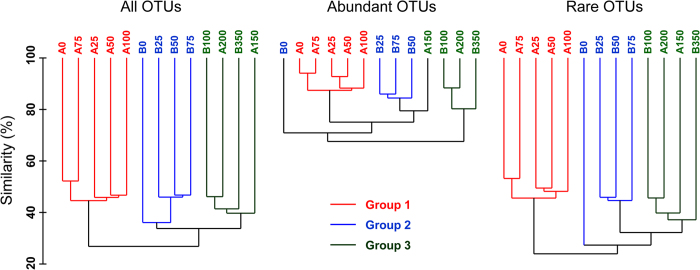
Cluster analysis of bacterial community based on Bray-Curtis similarity. The clustering pattern including all OTUs is compared with the clustering obtained for abundant OTUs only (relative abundance ≥1%) and for rare OTUs only (<0.01%). Colors highlight the clusters conserved through the three analyses. The letters A and B indicate stations A and B, and the numbers indicate sampling depths, respectively.

The high-throughput sequencing generated a total of 489,589 quality-filtered sequences, averaging 37,661 sequences per sample, which were classified into 8,977 distinct OTUs at 97% similarity level. The rarefaction analysis showed that bacterioplankton richness was satisfactorily captured within the whole set of OTUs from the 13 samples, although non-saturated curves from single samples would suggest that diversity was not entirely covered at a local scale (Supplementary Figure S1). Regardless of such intrinsic limitation when comparing differences in bacterial community composition within the 13 samples, we chose entire, abundant and rare OTUs for cluster analysis, showing that the three groups were distinguishable (Fig. 2). Comparing the unique and shared OTUs from two stations (at 3% distance threshold), 31.2% and 35.4% of OTUs were exclusively retrieved from stations A and B, respectively. Within the entire community, the dissimilarity between surface waters of Groups 1 and 2 was higher than that calculated among the deeper waters within the Group 3, thus meaning that distinct phylogenetic patterns differentiate the three identified groups (Fig. 2). According to the results of SIMPER analysis, the main members contributing to such differentiation were the OTU13 and OTU14 in Group 1 (13.77% and 13.25%), OTU3 in Group 2 (15.74%), OTU5 and OTU8 in Group 3 (17.83% and 13.07%) (Supplementary Figure S2). Our results further showed that the grouping for the abundant and rare subcommunities in general were similar to the clustering of the entire community (Fig. 2).

**Figure 3:**
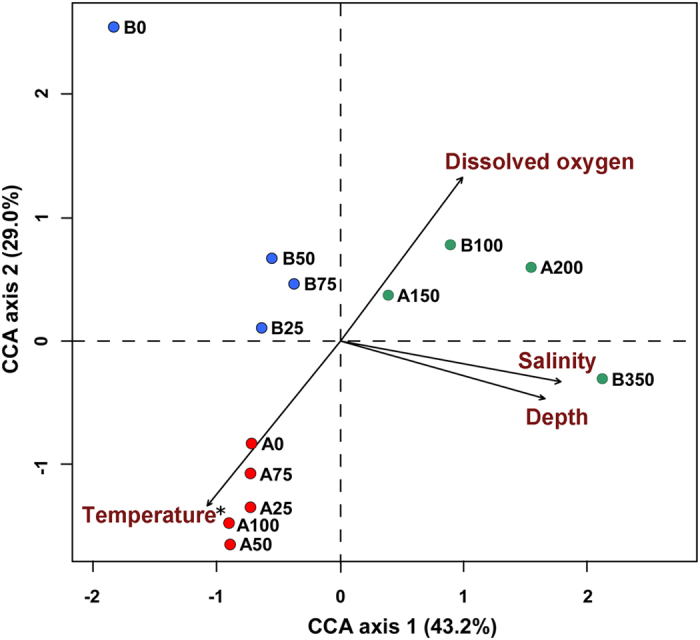
CCA ordination showing the effect of environmental factors on the bacterial community. The statistically significant variable is marked with an asterisk (*) according to a Monte Carlo permutation test (*P* < 0.05).

The result of CCA ordination showed that temperature was the significant environmental variable affecting bacterial community (Fig. 3). The first CCA axis was strongly correlated with salinity, depth, dissolved oxygen and temperature, whereas the second axis was related with dissolved oxygen and temperature.

**Figure 4:**
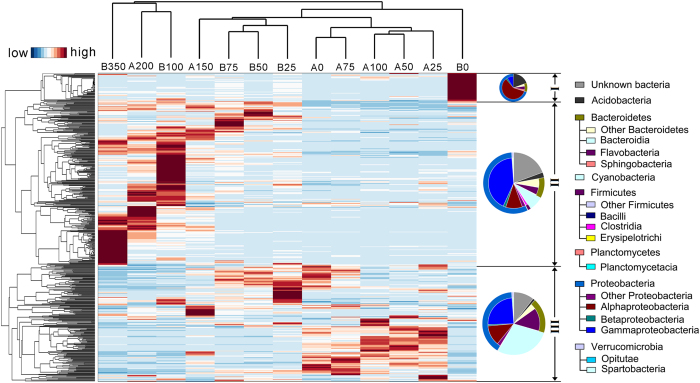
Heat map showing the relative abundance of selected abundant bacterial taxa. The microbial assemblage structure was determined using the V4 region of 16S rDNA barcodes. The relative abundance of OTUs is reflected by the color of scale. The 500 most abundant OTUs (3% distance) were clustered in R-mode, resulting in three groups (or branches), and the groups I, II, III were characterized by different taxonomic compositions from B0, mesopelagic samples and epipelagic samples except B0, respectively. The three pie plots represent the average abundance of the bacterial OTUs at both phylum and class levels across all samples from groups I, II, III, respectively.

**Figure 5:**
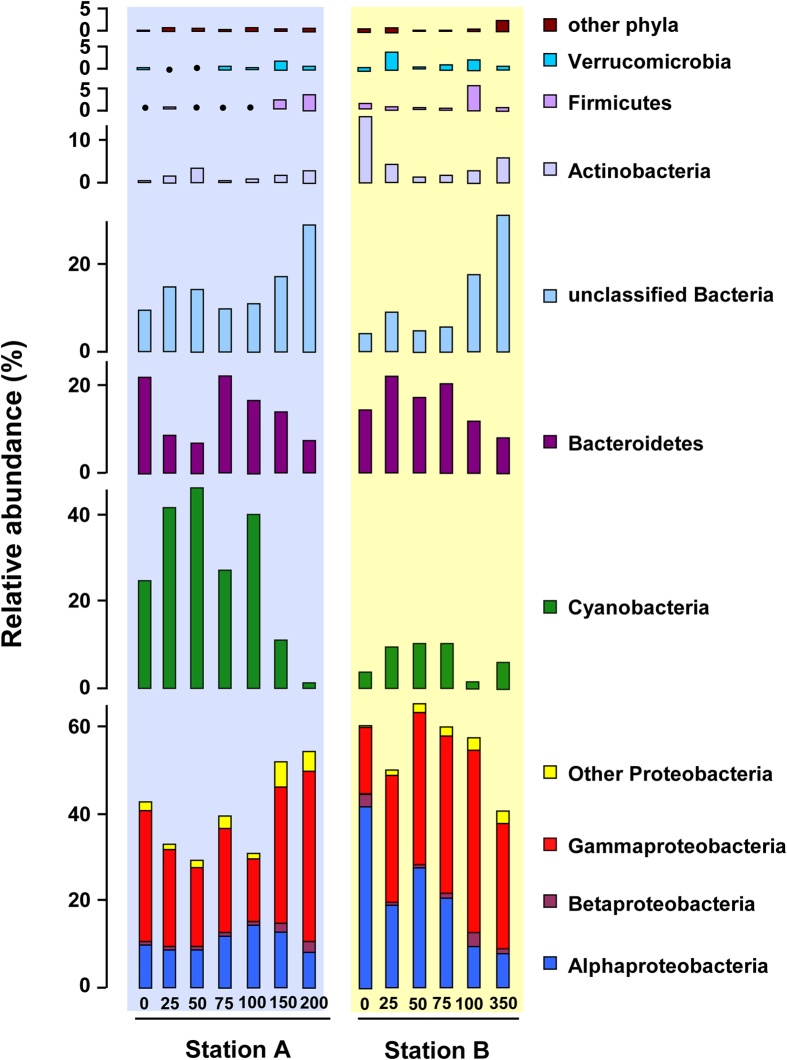
Phylum-level (and class-level for proteobacteria) assignments of the 16S rRNA gene sequences in two Southern Ocean stations.

The heat map clearly showed the distribution patterns of bacterioplankton communities between surface epipelagic and deep mesopelagic waters (Fig. 4). There were 7 main phyla and more than 12 main classes (OTU relative abundance >1% in a sample). Interestingly, a high abundance of Alphaproteobacteria was captured at B0, with a decreasing trend along the water column of station B. Gammaproteobacteria were mostly found in mesopelagic waters, whereas Flavobacteria (Bacteroidetes) were prominent in epipelagic waters. Proteobacteria were the overwhelmingly dominant taxa and four subdivisions of Proteobacteria were further recognized in all of the 13 samples (Fig. 5). Unclassified bacteria were significantly higher in the deep mesopelagic waters from both stations A and B. More importantly, there were more unclassified bacterial groups among the rare OTUs compared with abundant bacteria especially in the deepest waters. Cyanobacteria accounted for as much as 46.1% of total bacterial sequences in the surface waters (Group 1) of station A, but dropped to less than 1% in the deep water at depth 200 m.

To conduct a detailed analysis on dominant taxa, we defined the most dominant (17 in total) as those OTUs accounting for ≥5% of the sequences in at least one sample (Supplementary Figure S2). These top 17 OTUs together represented about 48% of the total sequences. Among those top 17 OTUs, OTU3, OTU5 and OTU6 were found in all samples, whereas both OTU7 and OTU16 were found in only one sample. Eleven of the top OTUs in both stations belonged to Gammaproteobacteria or Alphaproteobacteria. The relative abundance of Proteobacteria and Cyanobacteria OTUs differed significantly among the identified groups (*P* < 0.05). Cyanobacteria were among the dominant taxa especially at station A, and their abundance in upper layer waters was higher than in the deeper waters. Surprisingly, the OTU16, belonging to Actinobacteria, was only found in the surface sample of station B (Supplementary Figure S2).

## Discussion

Few reports have compared bacterioplankton communities at different water depths in the Southern Ocean^1^,^15^. In this study, most of the 16S rRNA gene copies were found in surface waters at 75 m, likely corresponding to the deep chlorophyll-a maxima previously reported for Antarctic waters^30^. Significant differences in bacterial communities were found between surface waters of stations A and B, whereas communities from deeper waters exhibited higher level of similarity, as it was assumed by the group definition (Fig. 2). Our CCA results indicated that water temperature was the most important factor driving the stratification of bacterial community structure. By affecting water density and, in general, the physical and chemical characteristics of different water masses, the temperature variations could have further contributed to niche differentiation in the water column. The geographical distance between the two stations (around 150 km) would not seem far enough to explain entirely the significant differences in taxonomic composition occurring among bacterial communities of surface waters from stations A and B (i.e., A0 and B0). Local physical and chemical factors could be important in controlling the occurrence and dominance of different bacterial OTUs in the photic open ocean in complex ways^5^,^15^, thus explaining at least in part the different diversity profiles found between the sampling locations (see Figs 3 and 4).

Distinct vertical distribution patterns of the bacterial communities were identified in the transition from epipelagic to mesopelagic waters. The surface community was dominated by few taxa with a high abundance, while the deep communities consisted of many taxa with lower abundance. These differences suggest a direct effect of vertical gradients along the water column^15^. Previous studies have found that microbial communities display vertical patterns in marine and freshwater ecosystems^31^,^32^. Piquet *et al.* suggested that melt water stratification and the transition to varying conditions in the Southern Ocean surface waters may have an impact not only on the micro-eukaryotic community but also on bacterial community composition^32^.

The predominance of both Alphaproteobacteria (including *Roseobacter* and SAR11 clades) and Gammaproteobacteria (including SAR86 clade) in terms of relative abundance and taxonomic richness is consistent with community patterns observed throughout the global ocean^33^,^34^. In our study, Cyanobacteria were among the dominant taxa especially in surface waters of station A (35%), and their abundance decreased with depth, as it is expected because of their property of photoautotrophic metabolism^35^. Interestingly, the lack of Cyanobacteria in surface waters of station B (only the 8% of total OTUs retrieved) corresponded to a high occurrence of sequences affiliated to Flavobacteria, at proportions much larger than those found at station A. The class Flavobacteria, belonging to the widespread phylum Bacteroidetes, was reported to play an important role in marine organic matter degradation^36^, and to profit from the co-occurrence of phytoplanktonic species^37^. Recently, Teeling *et al.*^38^ recorded a higher cell abundance of different species of Flavobacteria after phytoplankton bloom events. In line with such published findings, the different Cyanobacteria/Flavobacteria ratios between the sampling stations (e.g., 1.45 at A0 vs 0.50 at B0) further indicated that either abiotic (e.g., physical and chemical characteristics) or biotic factors (e.g., phytoplankton competition, zooplankton predation, virus parasitism) may crucially affect microbial distribution patterns in epipelagic waters at a local scale^34^,^35^,^39^,^40^.

Different selection and driving mechanisms for maintaining the surface and deep ocean community structure and diversity were identified^15^,^34^,^41^. Generally, bacterioplankton communities in surface waters are more governed by environmental selection, such as short-term and more variable conditions, whereas bacterial communities in deep waters are mostly shaped by longer water mass residence time and more stable environmental conditions, which are dependent on oceanic circulation and constrained by historical events^42^. How the diversity patterns of microbial communities reflect the environmental heterogeneity can be now explored in remote areas and at finer phylogenetic scale, as changes in microbial taxonomic composition can be measured with greater sensitivity owing to high-throughput sequencing methods. To date, our understanding of the distribution and role of microbes in the oceans has focused on abundant taxa, despite most of the marine bacterial species diversity is determined by low abundant or rare taxa^26^,^34^,^43^. The highly diverse and rare microbial biosphere remains largely unexplored^22^,^43^. This could partly explain the high proportion of unclassified bacteria, showing <50% similarity with the 16S rDNA deposited sequences, that was found in the deep mesopelagic waters of Southern Ocean. However, these unclassified bacteria should be treated with a little caution given the potential methodological bias owing to the short read length of Illumina fragments.

In summary, the application of molecular methods, including quantitative PCR and high-throughput sequencing, provided comprehensive and integrated information on the community composition and diversity patterns of bacterioplankton in two water columns of the Southern Ocean. The phylogenetic community composition was likely affected by the vertical environmental gradients occurring in the transition from epipelagic to mesopelagic waters. Surface communities were more dissimilar from their respective deep communities, which differed less and showed higher community diversity compared with the surface communities. This study shows that vertical stratification and local variations in the Southern Ocean influence the bacterioplankton communities along water column in complex ways, thereby indicating that epipelagic and mesopelagic ecosystems may hold two different mechanisms for the development and maintenance of microbial communities.

## Additional Information

**How to cite this article**: Yu, Z. *et al.* Bacterioplankton community shifts associated with epipelagic and mesopelagic waters in the Southern Ocean. *Sci. Rep.* **5**, 12897; doi: 10.1038/srep12897 (2015).

**Accession codes**: All sequence data from this study have been deposited in the public NCBI database (http://www.ncbi.nlm.nih.gov/) under the accession number SRX386611.

## Supplementary Material

Supplementary Information

## Figures and Tables

**Table 1:** Environmental and bacterial parameters in three groups from the two Southern Ocean stations.

Water layer	Group 1 (n = 5)	Group 2 (n = 4)	Group 3 (n = 4)
Temperature (°C)	0.52 ± 0.01^a^	0.12 ± 0.23^a^	−0.59 ± 0.22^b^
Salinity (‰)	34.30 ± 0.01^a^	34.28 ± 0.01^a^	34.47 ± 0.06^b^
Dissolved oxygen (mg l^−1^)	7.97 ± 0.01^a^	8.06 ± 0.05^ab^	8.20 ± 0.05^b^
Electrical conductivity (S m^−1^)	2.90 ± 0.01^a^	2.86 ± 0.02^ab^	2.82 ± 0.03^b^
Shannon-Wiener index	4.21 ± 0.06^a^	4.35 ± 0.11^a^	4.94 ± 0.04^b^
Number of OTUs	1746 ± 83^a^	1976 ± 101^a^	2228 ± 76^b^
Number of 16S rDNA (copy l^−1^)	(4.57 ± 2.25) × 10^10a^	(0.21 ± 0.14) × 10^10a^	(0.23 ± 0.10) × 10^10a^
Proteobacteria (%)	35.06 ± 2.61^a^	58.70 ± 3.34^b^	50.75 ± 3.69^b^
Cyanobacteria (%)	35.76 ± 4.24^a^	8.50 ± 1.61^b^	4.88 ± 2.37^b^
Bacteroidetes (%)	15.40 ± 3.25^a^	18.33 ± 1.65^a^	10.28 ± 1.48^a^
Unclassified bacteria (%)	11.88 ± 1.08^a^	5.93 ± 1.07^a^	25.13 ± 4.69^b^

Analysis of variance (ANOVA) was used in combination with Scheffe’s F multiple-comparison test to examine differences among the parameters of the three groups. Different lower case letters indicate significant differences among the groups.

Data represent mean ± SE.
