# Supplementary material for: Bacterioplankton community shifts associated with epipelagic and mesopelagic waters in the Southern Ocean
Source: Sci Rep. 2015 Aug 10;5:12897. doi: 10.1038/srep12897 (PMC4530437; doi:10.1038/srep12897)
Supplement: Supplementary Information [file srep12897-s1.doc]

Supplementary Information

**Bacterioplankton community shifts associated with epipelagic and mesopelagic waters in the Southern Ocean**

Zheng Yu ^1^, Jun Yang ^1^*, Lemian Liu ^1^, Wenjing Zhang ^2^ & Stefano Amalfitano ^3^

^1^Aquatic EcoHealth Group, Key Laboratory of Urban Environment and Health, Institute of Urban Environment, Chinese Academy of Sciences, Xiamen 361021, P. R. China;

^2^Marine Biodiversity and Global Change Center, College of Ocean and Earth Sciences, Xiamen University, Xiamen 361005, P. R. China;

^3^Water Research Institute, National Research Council of Italy (IRSA-CNR), Monterotondo, Roma, 00015, Italy.

*Correspondence: Jun Yang, Aquatic EcoHealth Group, Key Laboratory of Urban Environment and Health, Institute of Urban Environment, Chinese Academy of Sciences, Xiamen 361021, P. R. China. Tel/Fax: +86(0)592 6190 775; E-mail: [jyang@iue.ac.cn](mailto:jyang@iue.ac.cn)


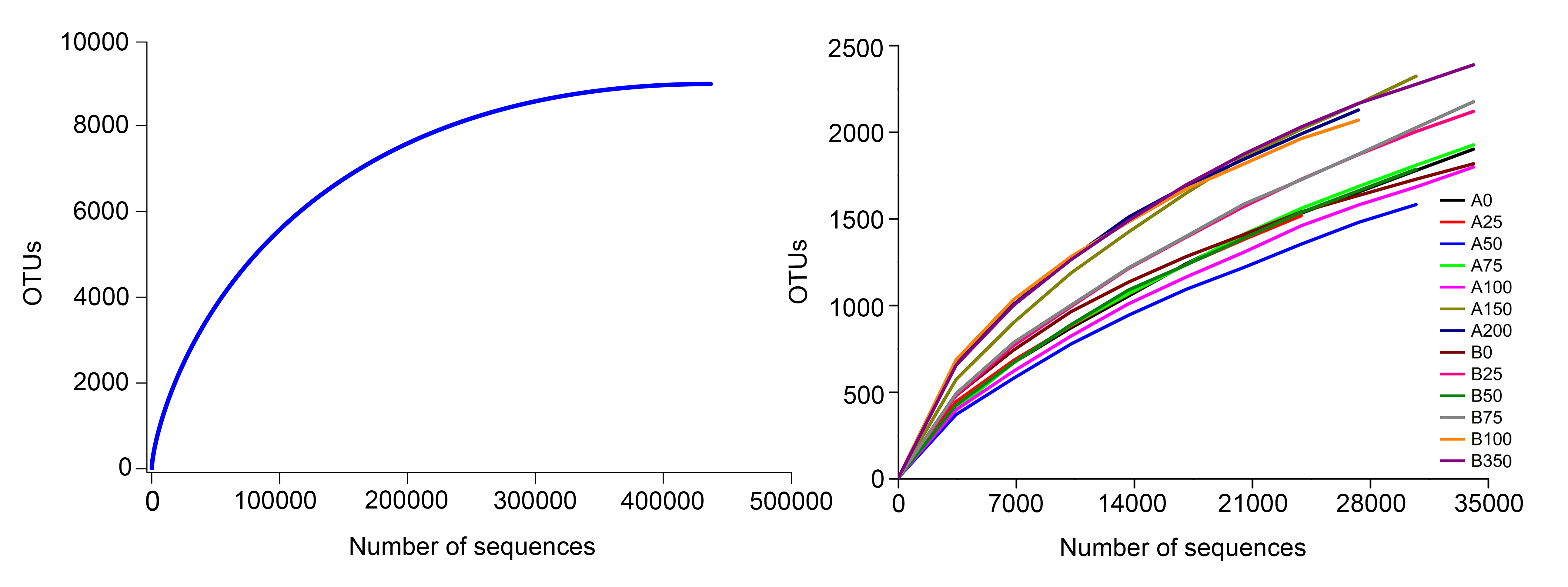


**Figure S1** Rarefaction curves of similarity-based operational taxonomic unit (OTUs) at cluster distance value of 0.03. Left – curve from the combined set of 13 samples, right – curves from individual samples.


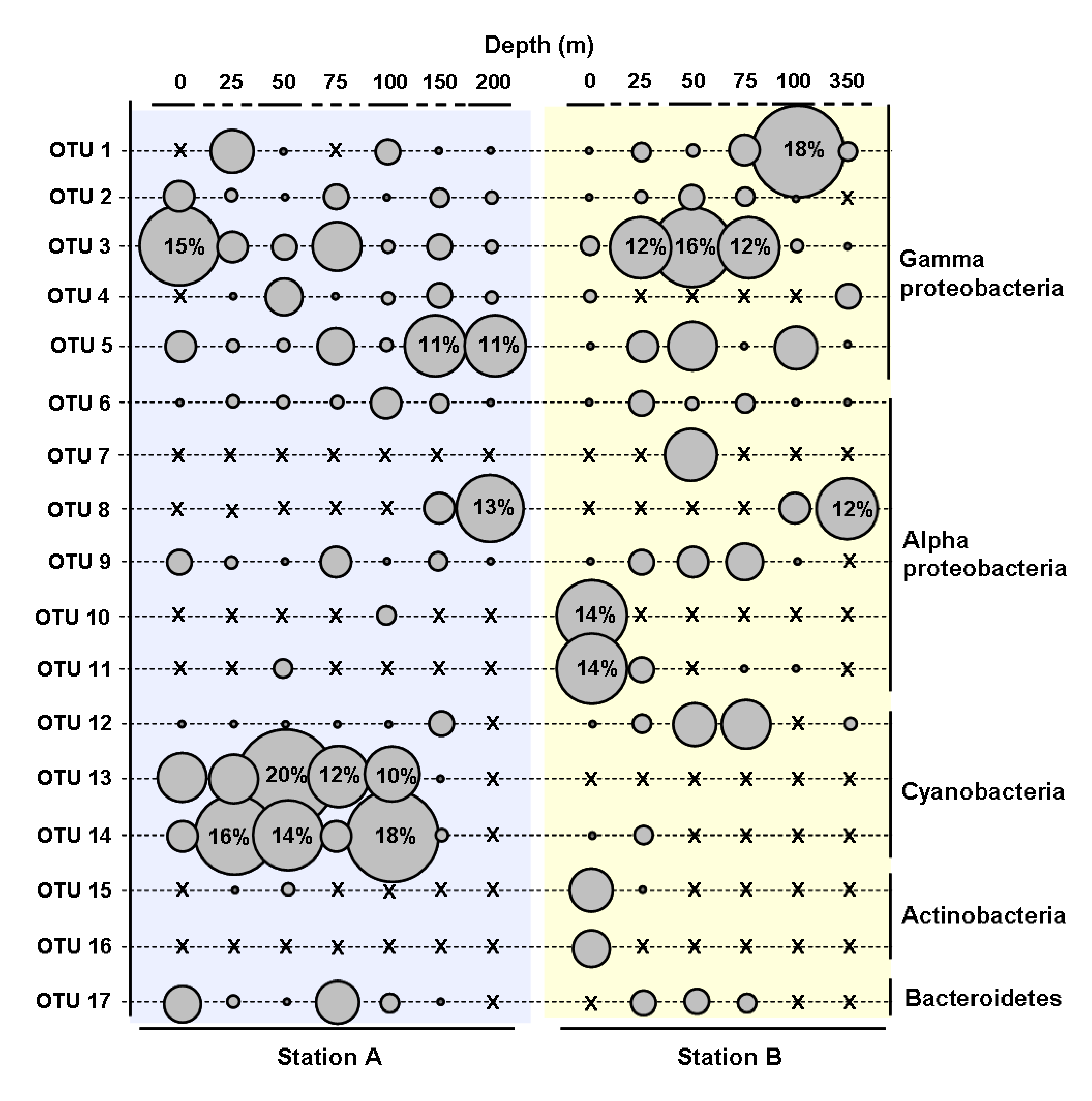


**Figure S2** Distribution of the dominant bacterial OTUs associated with the sampling sites and water depths. Shown are OTUs representing ≥ 5% relative abundance in at least one sample (totaling 17 OTUs). OTUs with relative abundances < 0.01% (rare OTUs in a fraction of the samples) are indicated with an x and the rest OTUs (≥ 0.01%) are indicated with a circle.
